# Numerical simulation of upper airway heat transfer in children with mandibular retrognathia during inspiratory process

**DOI:** 10.3389/fped.2023.1285812

**Published:** 2023-11-03

**Authors:** Yikai Gao, Hongyu Liu, Na Liu, Li Zhang

**Affiliations:** ^1^ Department of Ultrasound Intervention, Shandong Provincial Third Hospital, Shandong University, Jinan, China; ^2^ Department of Critical Care Medicine, Shandong Provincial Third Hospital, Shandong University, Jinan, China; ^3^ Department of Ultrasound Diagnosis and Treatment, The First Affiliated Hospital of Shandong First Medical University & Shandong Provincial Qianfoshan Hospital, Jinan, China

**Keywords:** airway, temperature, mandibular retrognathia, computational fluid dynamics, heat transfer

## Abstract

**Introduction:**

The human upper airway regulates temperature, but its heating capacity remains unclear when the ambient temperature is low and the airway structure is abnormal. Therefore, the purpose of this study was to evaluate the heat transfer characteristics of the upper airway in patients with mandibular retrognathia, and to quantitatively evaluate the influence of ambient temperature on the temperature field of the upper airway, which could provide a valuable reference for the prediction, diagnosis and treatment of respiratory tract related diseases.

**Methods:**

Two typical ambient temperatures of —10 °C and 20 °C were selected to numerically simulate the air flow and heat transfer synchronization in the upper airway model of mandibular retrognathia under quiet breathing and heavy breathing.

**Results and discussion:**

The results showed that the inspired air could not be sufficiently heated after flowing through the upper airway and main trachea in the two breathing states under low temperature conditions, and the inferior bronchus was more stimulated under the state of heavy breathing. In addition, the vortex flow structure in the upper airway can enhance the convective heat transfer effect in the corresponding area.

## 1. Introduction

Mandibular retrognathia is one of the common craniofacial deformities in Chinese children. Research has confirmed that children with mandibular retrognathia are more likely to cause obstructive sleep apnea hypopnea syndrome (OSAHS) than normal children (1, 2), which can cause signs such as growth retardation, hypertension, right heart failure, or pulmonary heart disease, seriously affecting the health of children (3, 4). The incidence rate of OSAHS in children can reach 1%–6% (5). The upper airway of human body serves as a channel for air exchange between the lungs and the external environment. It has important physiological functions such as filtering impurities, regulating temperature, humidity, and smell. Previous studies have shown that the clinical manifestations and severity of OSAHS in children vary greatly with seasons, and there is a great difference compared to adults, especially in winter (6). In addition, working in a cold environment may significantly affect cardiopulmonary function and tension levels (7), slowing down nerve conduction and muscle contraction (8). Therefore, it is necessary to study the heat transfer characteristics of upper airway in patients with mandibular retrognathia in different environments.

Changes in the anatomical structure of the upper airway can lead to changes in internal flow, which can affect heat transfer characteristics and temperature distribution. The distribution of airflow and temperature fields can directly reflect whether the physiological function of the upper airway is normal. Due to the complexity of the three-dimensional geometric structure of the upper airway and the difficulty in reaching its interior for detection, it is difficult to conduct quantitative analysis and research on the detailed temperature distribution inside the airway. The emergence of computational fluid dynamics (CFD) enables people to construct digital models to simulate the flow field inside the upper airway, thereby obtaining the distribution of the temperature field. Shamohammadi et al. studied the wall temperature distribution of the nasal cavity and trachea in an adult exposed to temperatures between 70 °C and 240 °C. It was found that the high temperature at the inlet dropped below 44 °C as it passed through the upper airway, and as the inlet temperature increased, the burned area gradually extended from the nasal cavity to the pharyngeal cavity (9). Issakhov et al. numerically simulated airflow transport in a nasal model of a healthy male under different environmental conditions to investigate the capacity of the nasal cavity to heat air during normal breathing at different temperatures (10). However, previous studies have mostly simplified the model or adopted individual healthy human models (11). Although the airflow inspired by normal people during quiet breathing can be heated to human body temperature when reaching the pharynx (10), the heat transfer characteristics of the upper airway, especially the pharyngeal cavity, are still unknown under the condition of abnormal upper airway anatomy and heavy breathing.

Therefore, this study intends to establish a three-dimensional model of a specific patient with mandibular retrognathia and conduct numerical simulation, which is crucial for studying the distribution of the temperature field in the human upper airway and recognizing the influence of upper airway morphology on heat transfer function.

## 2. Materials and methods

### 2.1. 3D model of upper airway

New Tom 5G CBCT was used to scan a patient with mandibular retrognathia under awake state. The patient was photographed in a supine position, with Frankfort plane perpendicular to the ground. The CBCT data was imported into Mimics 10.0 software (Materialise NV, Leuven, Belgium) to determine the three-dimensional coordinate direction of the image. Editing and reconstruction through five steps: (1) the upper airway threshold was set at −1024∼−289 HU; (2) The boundary of the upper airway was segmented; (3) Use the “regional growth” function to remove the disconnected structures around the upper airway; (4) Repair the upper airway boundary; (5) Finally reconstruct the 3D model.

### 2.2. Numerical simulation

The upper airway model was imported into Ansys workbench 19.2 (ANSYS, Inc, Canonsburg, USA) software, Fluent-Meshing was used for mesh, five boundary layers were set, the transition ratio was 0.272, and the growth rate was 1.2. Finally, an unstructured tetrahedral mesh was generated.

**Figure 1:**
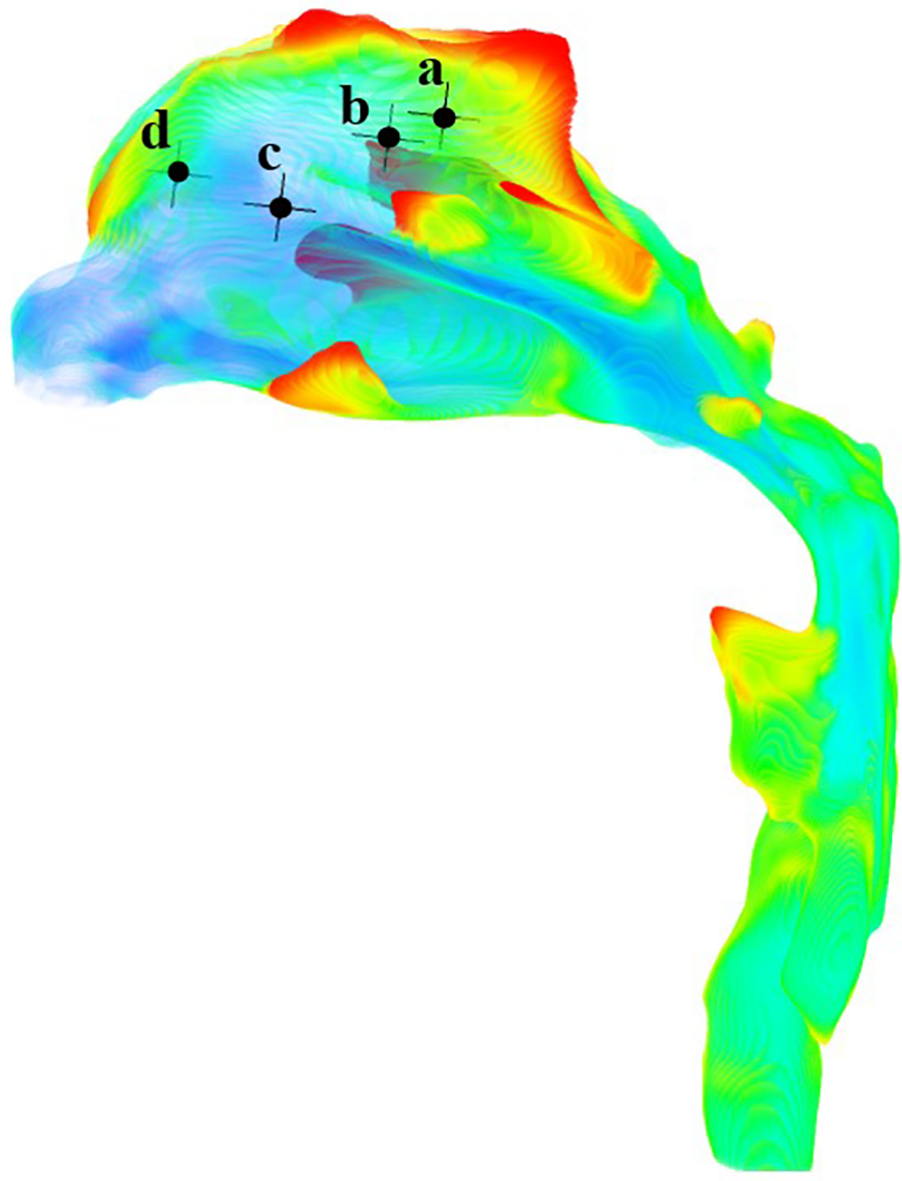
Location of four monitoring points in the olfactory region.

**Figure 2:**
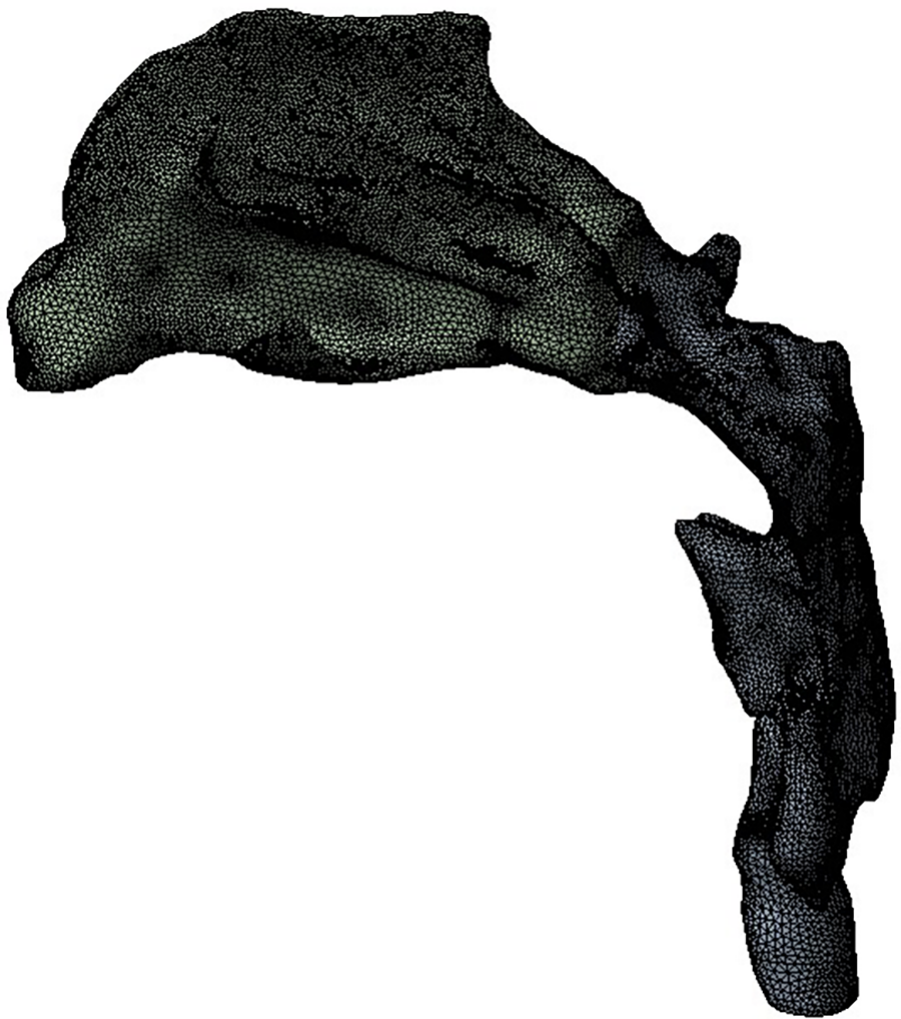
CFD meshing of upper airway model.

The anterior nostril was set to a standard atmospheric pressure. The temperature at the boundary of the anterior nostril was set at—10 °C and 20 °C (12), respectively. Assuming a free flow temperature of 37 °C (10), the convective heat transfer coefficient was 30 W/m^2^°C (13). The lower margin of the laryngeal cavity was set as the velocity inlet which changes sinusoidally with time. The tidal volume during quiet breathing was 180 ml and the inspiratory period is 2 s. 1100 ml for heavy breathing (14), and the inspiratory period was 1 s. The time step for quiet breathing conditions was 0.02 s, while the time step under heavy breathing condition was 0.01 s. Therefore, four cases were formed. In order to determine the optimal number of grids, we conducted a numerical simulation of the heat transfer characteristics of the upper airway during quiet breathing at an atmospheric temperature of −10 °C. Four monitoring points in the olfactory region of the nasal cavity (Figure 1) were selected. The relative temperature differences at the peak inspiratory moment were compared using 550,000, 730,000, and 970,000 grids, with 1.34 million grids as the standard (Table 1). It can be seen that the temperature of the monitoring point calculated using 970,000 grids was close to the calculation result of 1.34 million grids. This indicates that the temperature calculation is grid-independent. Therefore, the grid size of 970,000 was chosen for the calculations in this paper (Figure 2).

**Table 1:** Grid independence test at peak inspiratory moment.

Flow variables	Monitoring point	Relative difference (%)			
		550000 grids	730000 grids	970000 grids	1340000 grids
Temperature	a	8.45	6.02	1.44	/
	b	8.14	5.58	1.67	/
	c	7.85	5.72	1.58	/
	d	7.98	5.88	1.49	/

The SST k-ω turbulence model was used (15), and the pressure and momentum were discreteted in second order using the semi-implicit method of the pressure coupling equation. Normal air was used as the flow medium in the upper airway, and its physical characteristic parameters were as follows: *ρ *= 1.225 kg/m^3^; *k *= 0.0242 W/(m•K); and the thermal conductivity is the same in all directions.

### 2.3. Validation of CFD numerical models

**Figure 3:**
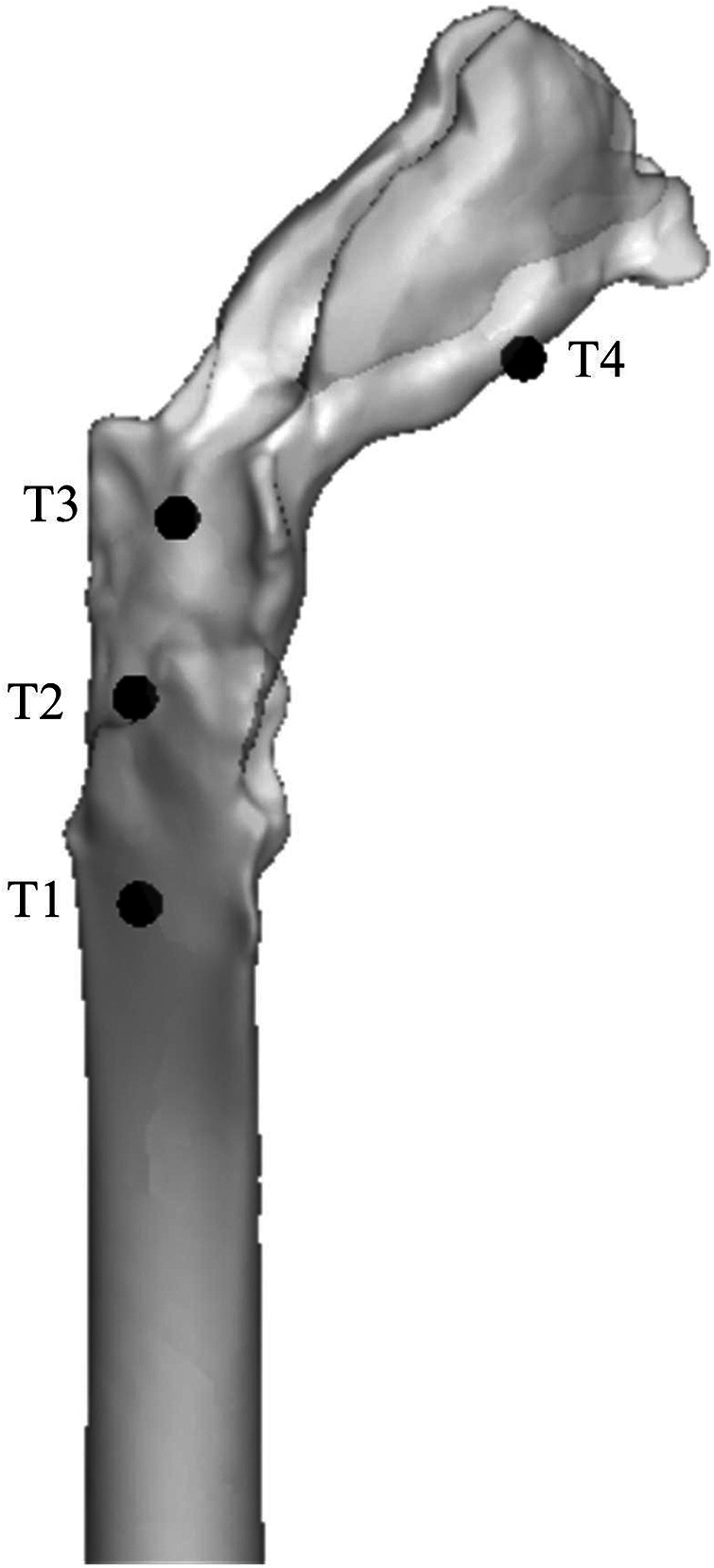
In vitro experimental airway model and location of pressure monitoring points.

A real human-scale upper airway model was tested *in vitro* using the experimental equipment established in previous studies (16). At a flow rate of 650 ml/s, the pressure of four pressure taps on the upper airway wall, simulated by CFD, was compared with that measured *in vitro* (Figure 3). The results showed that the CFD calculation results of the SST k-*ω* turbulence model were in good agreement with the experimental data (Table 2).

**Table 2:** Comparison of flow static pressures in the upper airway model predicted by CFD and measured in experiments (unit: Pa).

Pressure taps	Inspiration		
	CFD	Exp.	Difference (%)
T1	−28.53	−34.68	17.73
T2	−26.12	−30.63	14.72
T3	−23.50	−28.10	16.37
T4	−25.60	−29.67	13.72

## 3. Results

### 3.1. Temperature distribution of upper airway

Cross sections were established for the anterior, middle and posterior segments of the nasal cavity, as well as nasopharynx, oropharynx and trachea.

**Figure 4:**
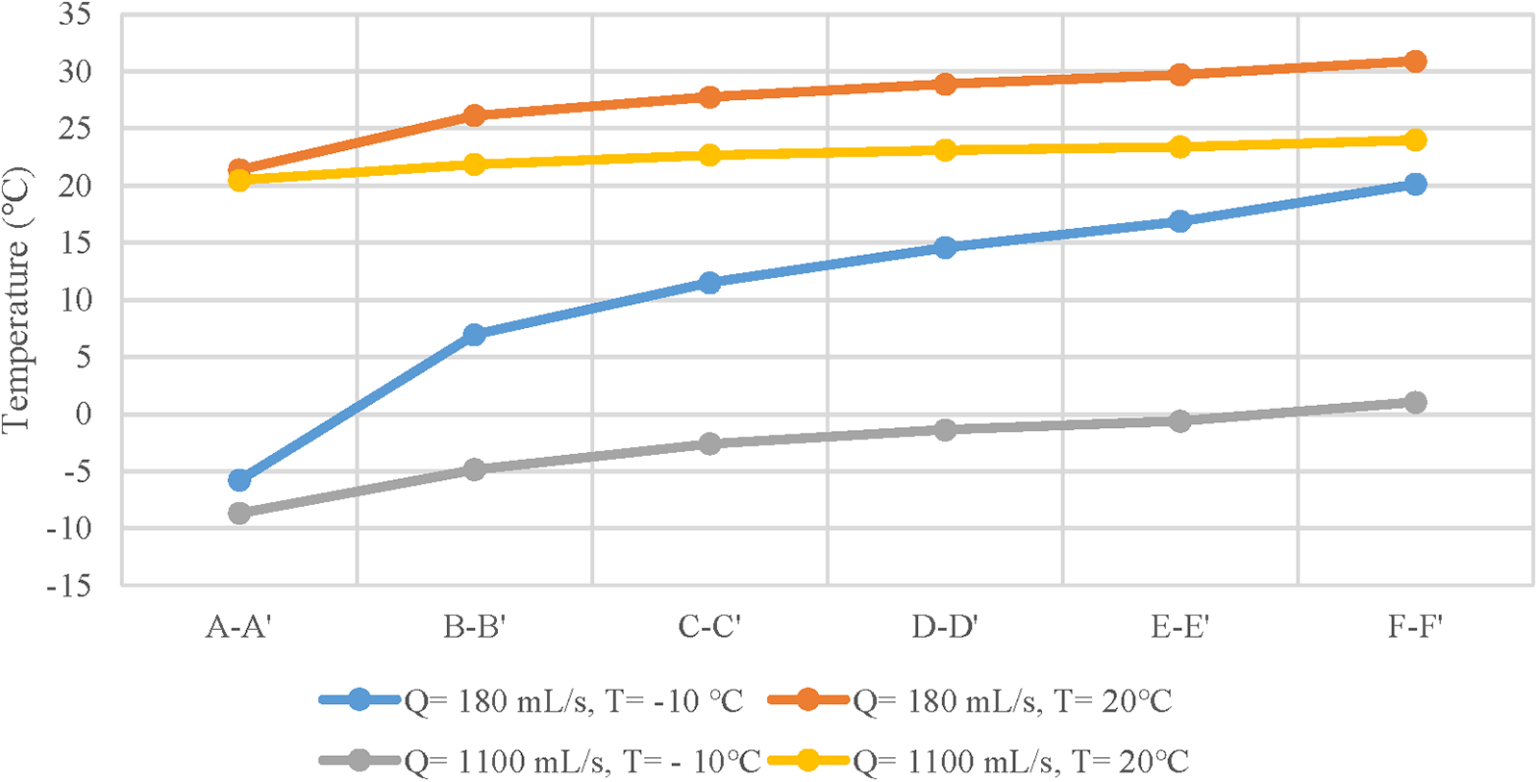
Temperature distribution curves of different anatomical sites at ambient temperatures of - 10 and 20 °C and tidal volumes of 180 and 1100 mL/s.

At −10 °C, for peak inspiratory moments of quiet breathing and heavy breathing, when cold air passes through the nasal cavity, the temperature increases to 11.50 °C and −2.61 °C, respectively. At 20 °C, the nasal temperature rises to 27.78 °C and 22.67 °C respectively at the above flow rate. This warming continues at a relatively steady trend in the nasopharynx and oropharynx until the trachea reaches 30.90 °C and 23.99 °C (Figure 4).

**Figure 5:**
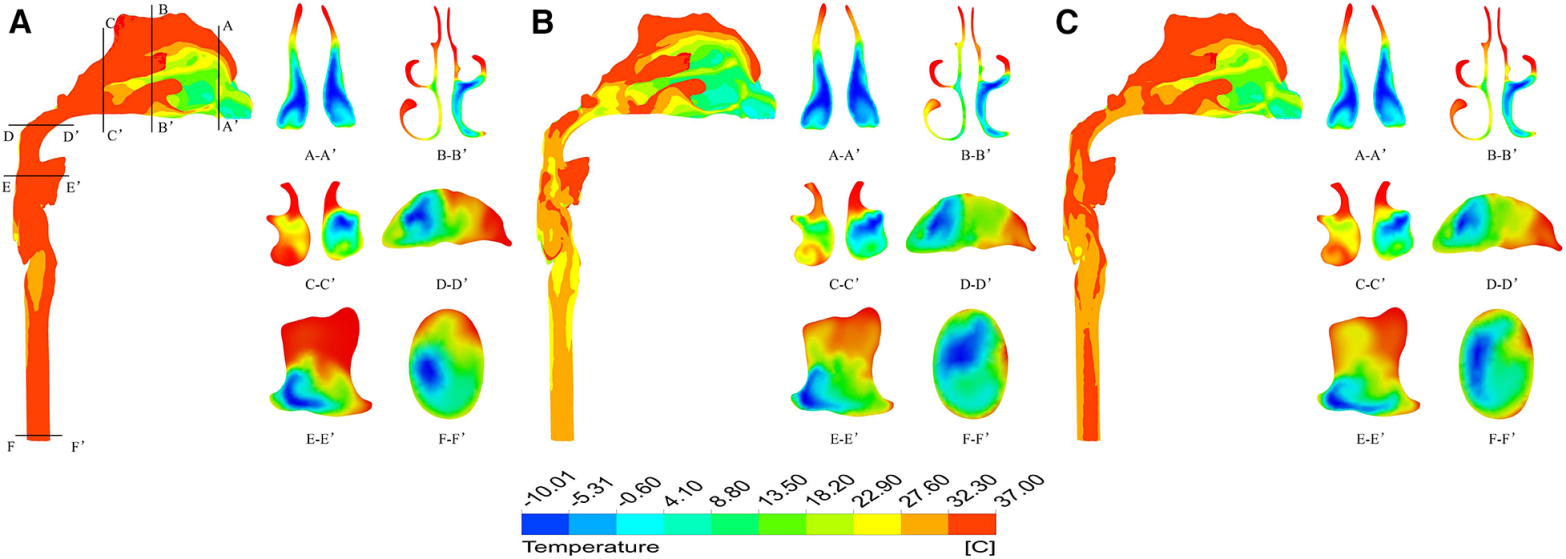
Temperature distribution at different times under quiet breathing state with ambient temperature of - 10 °C. (**A**) 0.4 s; (**B**) 1.0 s; (**C**) 1.6 s.

**Figure 6:**
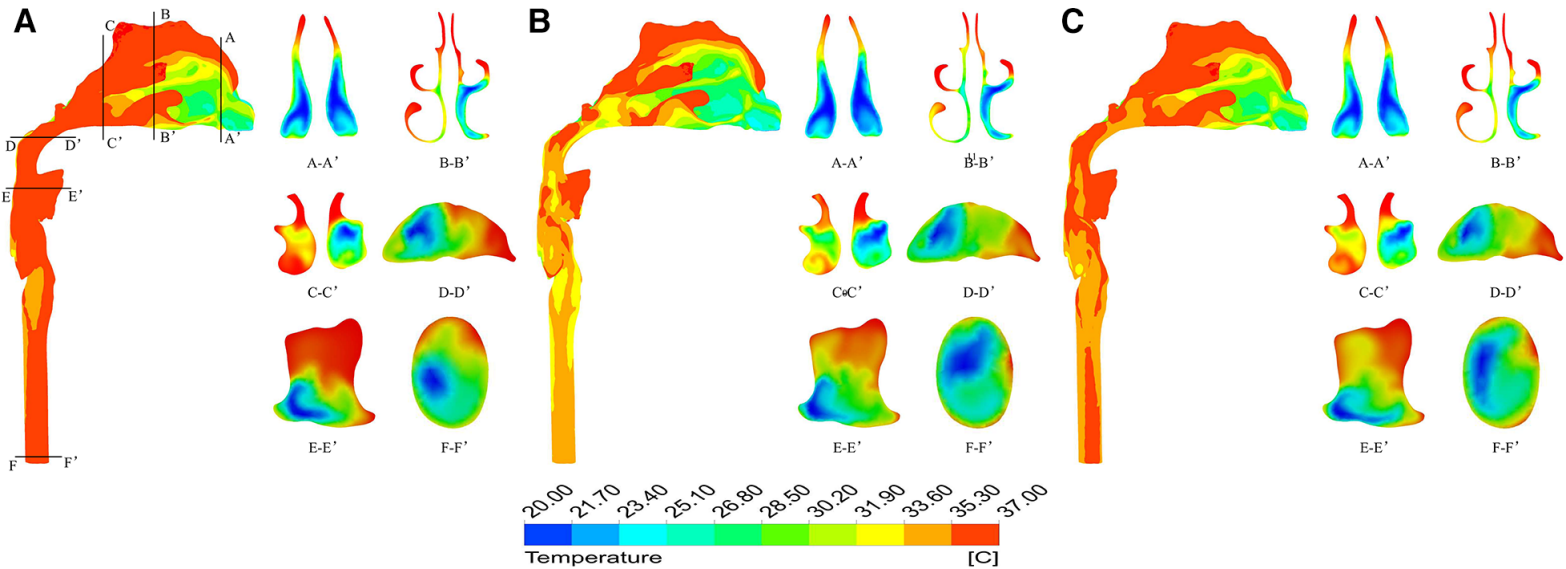
Temperature distribution at different times under quiet breathing state with ambient temperature of 20 °C. (**A**) 0.4 s; (**B**) 1.0 s; (**C**) 1.6 s.

Figure 5 shows the temperature contour at different inspiratory moments at −10 °C. 2103;. It can be found that the upper airway airflow temperature gradually increases from the anterior nostril to the laryngeal cavity during quiet breathing. From the beginning of inspiration to the peak moment of inspiration, the overall temperature of the upper airway flow decreases as the inlet velocity of the nasal cavity increases. Air heating was best in the olfactory fissure, middle nasal passage, and inferior meatus, while the air temperature was lower in areas near the middle of the nasal section. The upper airway air temperature increased significantly from the peak to the end of inspiration. When the ambient temperature was 20 °C and the breathing condition was quiet, the flow temperature field of the upper airway was basically consistent with that of the breathing condition at low temperature, except that the temperature increased somewhat (Figure 6).

**Figure 7:**
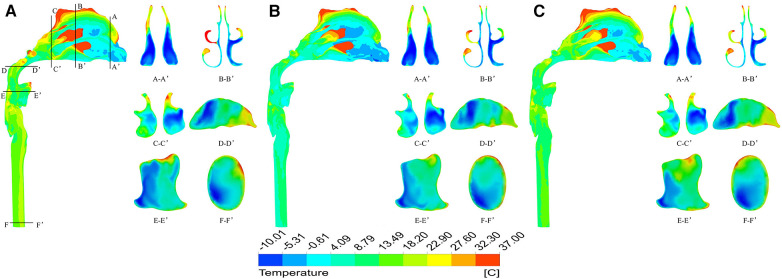
Temperature distribution at different times under heavy breathing state with ambient temperature of - 10 °C. (**A**) 0.2 s; (**B**) 0.5 s; (**C**) 0.8 s.

**Figure 8:**
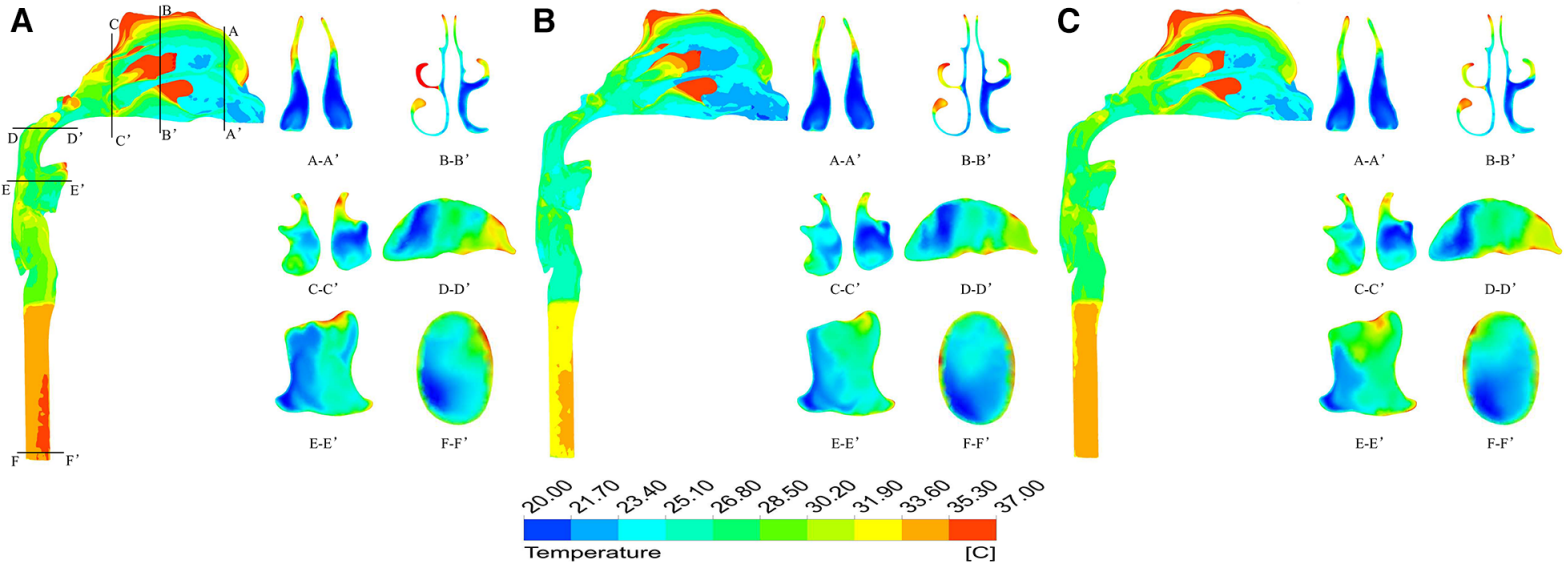
Temperature distribution at different times under heavy breathing state with ambient temperature of 20 °C. (**A**) 0.2 s; (**B**) 0.5 s; (**C**) 0.8 s.

The temperature field of the upper airway was significantly affected by breathing state. When the ambient temperature was −10 °C, taking the moment of maximum inspiratory velocity as an example, it can be found that under heavy breathing, the temperature of each section decreases significantly. The air flow temperature in a large area of the nasal front range was −10.0–18.7 °C, the lowest temperature at the nasal outlet was −8.1 °C, the average temperature was only −0.87 °C, and the average temperature at the laryngopharynx section was 0.75 °C (Figure 7). When the ambient temperature was 20 °C, the heating capacity of the upper airway also decreased under heavy breathing, but the temperature variation was not large due to the high temperature of the inspired air (Figure 8).

### 3.2. Temperature efficiency at each section of the upper airway

To quantitatively evaluate the heating degree of the air flow at each section of the upper airway, the following formula was used to represent the ratio of the actual heat absorption of cold air when it reaches the selected section to the heat absorption when it is fully heated.

(1) $$P=\frac{t_{\mathrm{ave}}-t_{0}}{t_{c}-t_{0}}$$

Where, *t_ave_* is the average temperature of the cross-section (mass weighted average), *t*_0_ is the ambient temperature, and *t_c_* is the core temperature of the human body, namely 37 °C.

It can be seen that at the moment of maximum velocity (Table 3), the heating degree of the inspired air at each cross-section was related to the breathing state, but not to the ambient temperature. The heating degree of air arriving at the same position in the state of quiet breathing was significantly higher than that in the state of heavy breathing. However, no matter in the state of quiet breathing or heavy breathing, the heating degree of each section before the inspired air flows through the trachea was not high, lower than 0.6.

**Table 3:** Temperature efficiency of each section of the upper airway during quiet and heavy breathing (quiet breathing t = 1 s, heavy breathing t = 0.5 s).

Breathing state	Ambient temperature	A-A’	B-B’	C-C’	D-D’	E-E’	F-F’
Quiet breathing	−10 °C	0.090	0.361	0.457	0.523	0.572	0.641
	20 °C	0.082	0.361	0.457	0.523	0.572	0.641
Heavy breathing	−10 °C	0.028	0.110	0.157	0.183	0.200	0.235
	20 °C	0.028	0.110	0.157	0.183	0.200	0.235

### 3.3. Relationship between temperature field and airflow field

Taking the numerical results of a quiet breathing state with an ambient temperature of −10 °C as an example, the velocity and temperature fields in the upper airway at the peak inspiratory moment were compared and analyzed.

**Figure 9:**
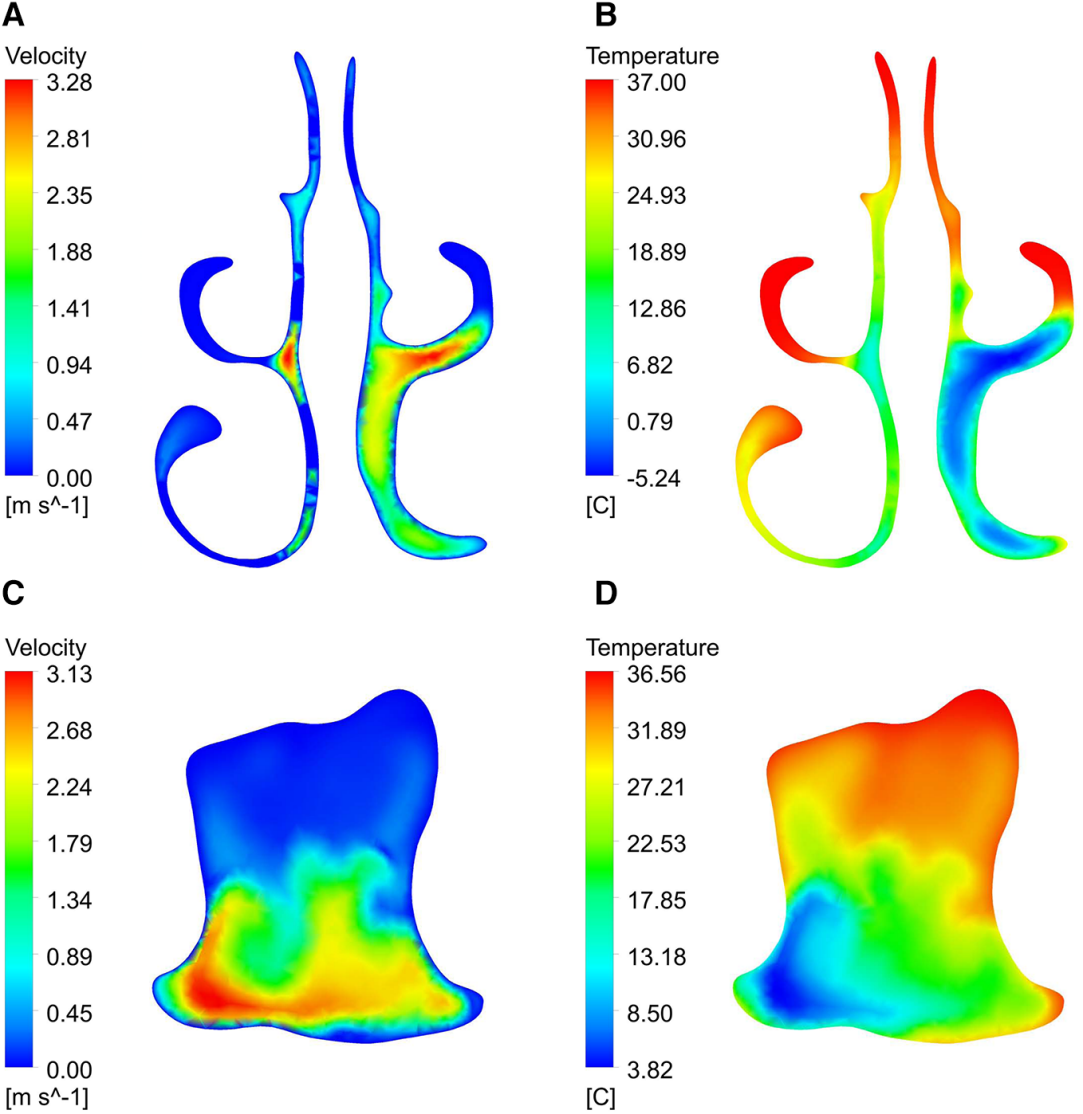
Velocity and temperature contour of section B-B ‘and section E-E’.

By observing the velocity and temperature contour of the two cross sections shown in Figure 9, it is found that the airflow velocities of the lower meatus, the middle meatus, the olfactory fissure area, and the front end of the oropharynx were significantly lower than those of the common meatus and posterior segment of the oropharynx, respectively.

**Figure 10:**
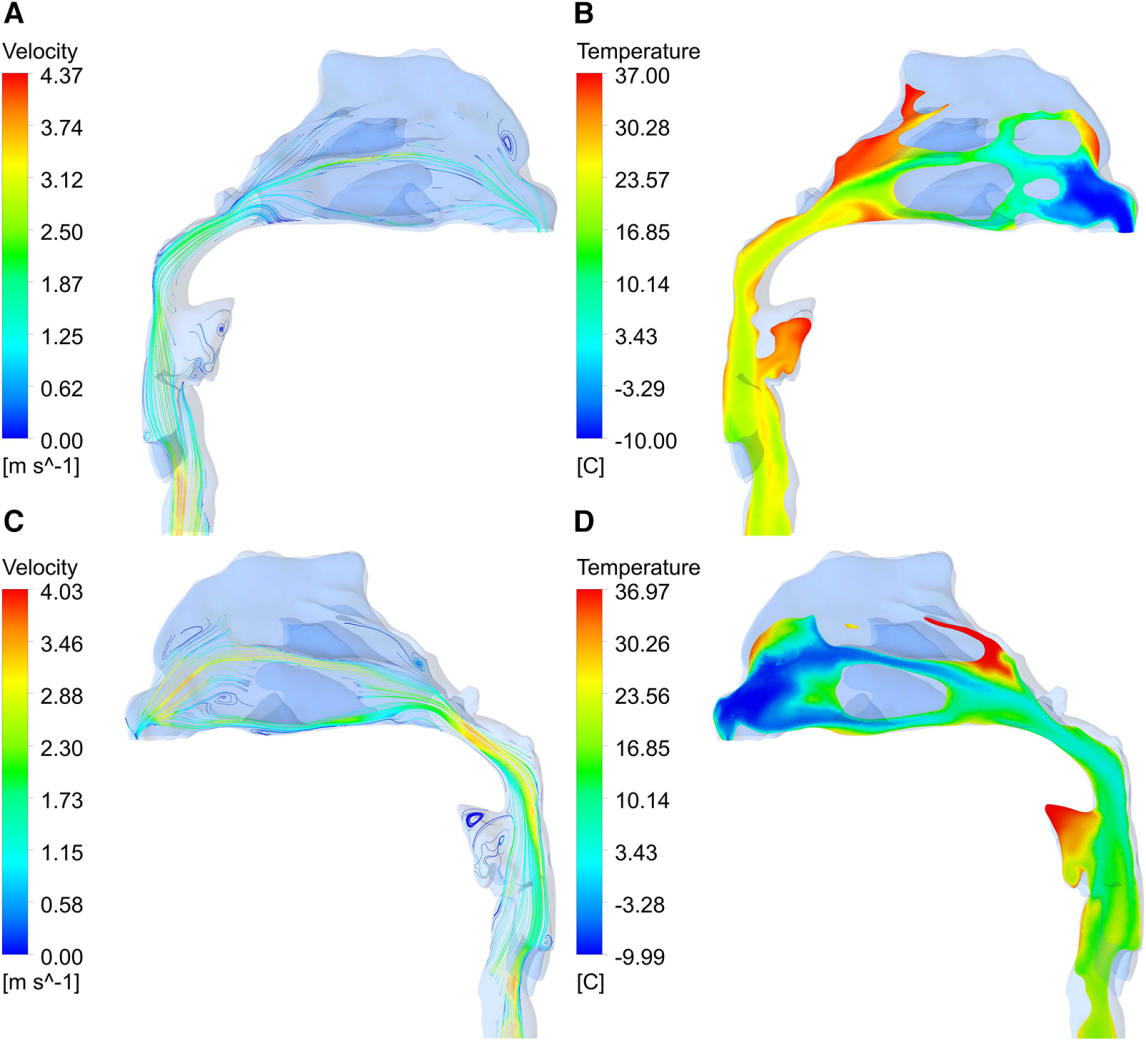
Velocity vector diagram and temperature contour of upper airway. (**A**, **B**) right section; (**C**, **D**) left section.

As can be seen from the profile of the upper airway (Figure 10), when the airflow flows through the nasal vestibule, due to the narrow passage, the velocity is relatively high. After reaching the pharynx, the cross-section of the airway shrinks, resulting in a rapid increase in velocity. In addition, influenced by the airway structure, there are many vortices of different sizes in the flow field, such as the top of the nasal vestibule and the bottom of the nasal outlet in Figure 10A, and at the front of the oropharynx in Figure 10C. By comparison with the temperature contour, it is found that the temperature of the airflow in the regions with vortices was generally higher than other locations on the same cross-section.

## 4. Discussion

Respiratory diseases often occur in winter (17, 18). Therefore, this paper focused on the study of heat transfer and flow characteristics in the human upper airway after inspiring cold air through the CFD numerical simulation method, and comprehensively evaluated the velocity field and related temperature distribution based on different respiratory flow and rates.

It is clearly from Figure 4 that the temperature distribution curves of the nasal vestibule and nasal cavity have the steepest slopes. This rise suggested that these anatomical sites balance the inspired air and the internal conditions of the body were remarkably efficient, and almost unaffected by the state of the surrounding air (10). This may be due to the complex geometry inside the nasal cavity, where most of the heat transfer process occurs, making the temperature of the inspired air closer to the operating temperature of the lungs (19). In addition, for a constant inlet temperature, an increase in inlet velocity can lead to deeper penetration of cold air into the respiratory system, resulting in more heavy damage in the respiratory system (11).

Convective heat transfer is strongly dependent on fluid velocity (20). The airflow velocity was not high in the olfactory fissure area, the middle meatus and the inferior meatus, so the air heating effect was better here (9). When airflow passed through narrow areas of the nasopharynx and oropharynx, the airflow velocity increased rapidly, so that this area was more affected by airflow temperature than its adjacent area. A strong vortex region was visible in the oropharynx, which increased the heat exchange between the airflow and the oropharynx wall (10, 11). Besides, due to the presence of vortices, a more turbulent flow is generated, which leads to an increase in heat transfer with the airway surface (15).

Under the same breathing state, even if the cold air temperature difference between cases is 37 °C, the temperature distribution of both still showed similar patterns during the inspiratory process. Compared with the quiet breathing state, the heating capacity of the upper airway to the air was greatly reduced in the heavy breathing state, and the cold air stimulated the respiratory tract more in the heavy breathing state. Therefore, the temperature distribution of the airflow depended on the respiratory velocity distribution at the inlet, rather than the temperature difference between the air and the upper airway surface (15).

The analysis of temperature efficiency and temperature at the outlet of the upper airway showed that when the human body was exposed to a low temperature environment in winter, whether in a state of quiet breathing or heavy breathing, the air inspired by patients with mandibular retrognathia could not be sufficiently heated to 37 °C after flowing through the upper airway and the main trachea, which can further stimulate the inferior bronchus. Therefore, the change of upper airway geometry was also an important factor affecting the distribution of internal temperature field, and long-term exposure to low temperature environment may easily cause respiratory diseases.

There are still some limitations in this study. One is that when studying the heat transfer characteristics of the upper airway, its humidification ability to the air is ignored, and the influence of humidity on heat transfer is not considered; Second, the boundary conditions are simplified, and the heat transfer between blood and mucus layer is ignored, resulting in a certain deviation between the numerical simulation results and the actual situation.

## 5. Conclusion

Breathing state has a significant impact on the temperature field of the upper airway. Compared to quiet breathing state, heavy breathing significantly reduces the heating capacity of the upper airway to air, and cold air has greater irritation to the respiratory tract under heavy breathing state.

## Data Availability

The original contributions presented in the study are included in the article/Supplementary Material, further inquiries can be directed to the corresponding author.
